# Pregnancy-induced Cushing’s syndrome with an adrenocortical adenoma overexpressing LH/hCG receptors: a case report

**DOI:** 10.1186/s12902-020-0539-0

**Published:** 2020-05-11

**Authors:** Shaohua Li, Chen Yang, Jing Fan, Yao Yao, Xiaomei Lv, Ying Guo, Shaoling Zhang

**Affiliations:** Department of Endocrinology, Sun Yat-sen Memorial Hospital, Sun Yat-sen University, 107 Yanjiang West Road, Guangzhou, 510120 China

**Keywords:** Cushing’s syndrome, Pregnancy, LHCGR, Cyclic adenosine monophosphate (cAMP) signaling pathway

## Abstract

**Background:**

Pregnancy-induced Cushing’s syndrome (CS) with an adrenocortical adenoma overexpressing luteinizing hormone (LH)/human choriogonadotropin (hCG) receptors (LHCGR) has been rarely reported in the literatures. This peculiar condition challenges the canonical diagnosis and management of CS.

**Case presentation:**

A 27-year-old woman (G2P0A1) presented at 20 weeks gestational age (GA) with overt Cushingoid clinical features. Adrenocorticotropic hormone (ACTH)-independent CS was diagnosed based on undetectable ACTH and unsuppressed cortisol levels by dexamethasone. Magnetic resonance imaging (MRI) scanning without contrast revealed a left adrenal nodule while pituitary MRI scanning was normal. A conservative treatment strategy of controlling Cushingoid comorbidities was conducted. At 36 weeks GA, a caesarean operation was performed and a live female infant was delivered. At 8 weeks after parturition, our patient achieved normalization of blood pressure, blood glucose, serum potassium, and urinary cortisol level spontaneously. During non-pregnancy period, stimulation testing with exogenous hCG significantly evoked a cortisol increase. The woman underwent resection of the adrenal tumor at 6 months after parturition. Immunohistochemistry (IHC) showed the tumor tissue that stained positive for luteinizing hormone (LH)/human choriogonadotropin (hCG) receptor (LHCGR), whereas negative for both melanocortin 2 receptor (MC2R) and G protein-coupled receptor-1 (GPER-1).

**Conclusions:**

Stimulation test with exogenous hCG after parturition is necessary for the diagnosis of pregnancy-induced CS. LHCGR plays an essential role in the pathogenesis of this rare condition.

## Background

Pregnancy-induced Cushing’s syndrome (CS) is rare, with a total of 15 cases reported in the world literature to our knowledge [1–15], and only two of those patients were secondary to an adrenal adenoma overexpressing luteinizing hormone (LH)/human choriogonadotropin (hCG) receptors (LHCGR) [12, 14]. Recently, hCG-dependent LHCGR activation coupled to cyclic adenosine monophosphate (cAMP) pathway has been considered to play an important role in pregnancy-induced CS [14, 15]. In 35–65% CS patients with cortisol-producing adrenal adenomas (CPA), somatic mutations of cAMP dependent protein kinase A (PRKACA) has been identified to constitutively activate cAMP/protein kinase A (PKA) pathway [16, 17]. We herein report a new case of pregnancy-induced CS with an adrenocortical adenoma. Considering that elevated estrogen during pregnancy could also potentially contribute to the activation of cAMP pathway through reacting on corresponding G-protein-coupled receptor (GPCR), immunohistochemistry (IHC) was performed on the adrenal adenoma tissue of our case to detect the expression of G protein-coupled receptor-1 (GPER-1), as well as LHCGR and melanocortin 2 receptor (MC2R). On the basis of previous findings and our IHC results, we discuss the underlying pathogenesis of this rare condition.

## Case presentation

A 27-year-old woman (G2P0A1) presented at 20 weeks gestational age (GA) with overt Cushingoid clinical features and was transferred to our hospital at 28 weeks GA. On physical examination, she had moon facies, acne, dorsocervical fat pad, violaceous striae and edema of both lower limbs. She was found to be hypertensive with a blood pressure of 140–150/70–90 mmHg. Laboratory examinations revealed hypokalemia with repeated low serum potassium measurements of 2.36, 2.99 and 3.13 mmol/L (reference range 3.5–5.5 mmol/L), and evidence of gestational diabetes mellitus (GDM) with a fasting plasma glucose of 7.24 mmol/L (reference range < 5.1 mmol/L) and oral glucose tolerance test (OGTT) 2 h plasma glucose of 12.27 mmol/L (reference range < 8.5 mmol/L). The menarche had occurred at age 16, and her menstrual cycles were irregular, occurring every 36 days, with moderate menstrual flow, lasting for 8 to 9 days. Her last pregnancy ended up with an abortion, but no Cushingoid symptoms or signs were observed and she was not diagnosed GDM or hypertension during prior pregnancy. Her past history was negative for hypertension, diabetes and hypercortisolism, but her father suffered from hypertension.

**Fig. 1:**
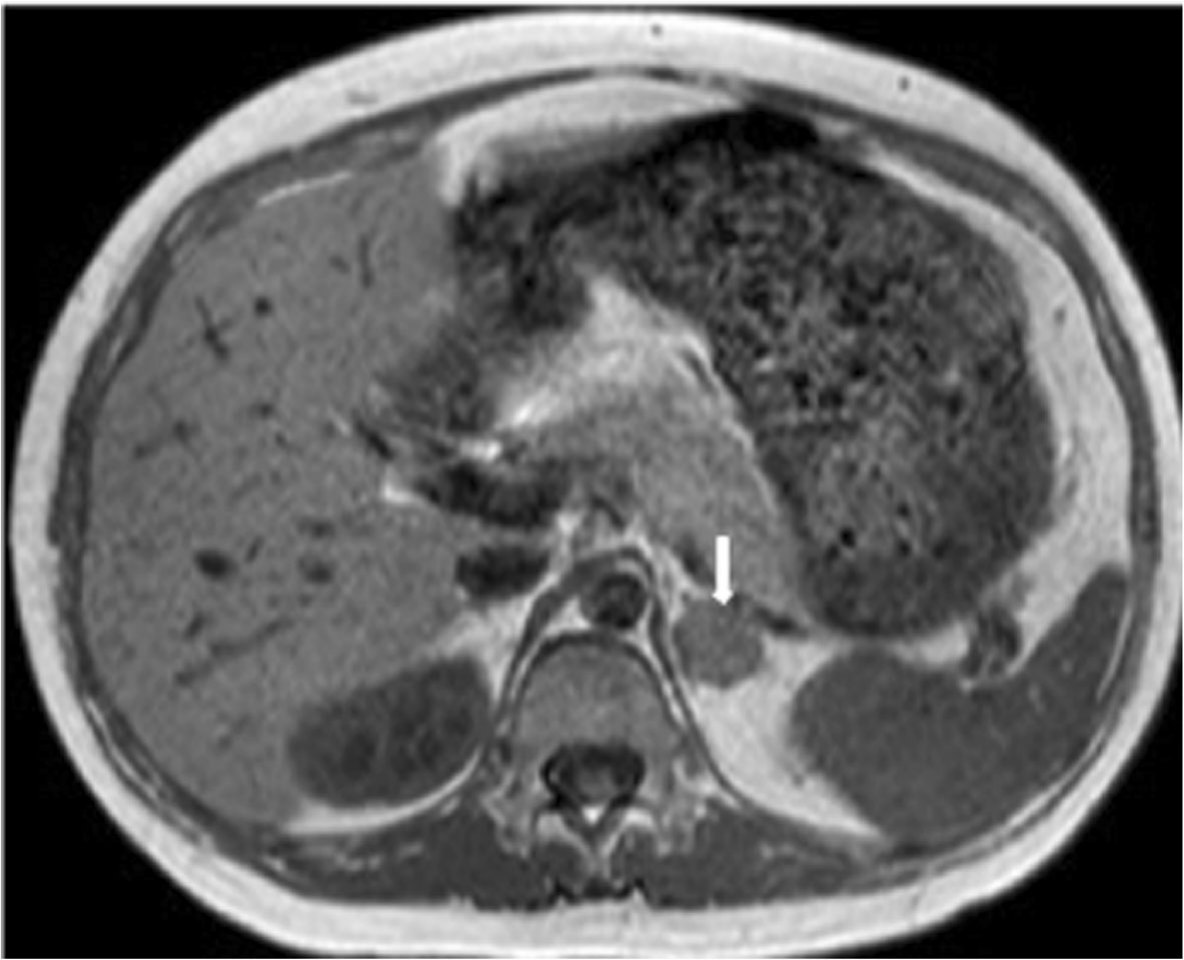
Adrenal magnetic resonance image (MRI) without contrast of the patient. Adrenal MRI showed a left adrenal nodule measuring 2.0 cm in diameter (arrow)

Preliminary examinations indicated hypercortisolism, including multiple elevated 24 h free urinary cortisol (24 h UFC) measurements of 2611.0 and 2590.0 nmol/24 h (reference range 153.2–789.4 nmol/24 h), midnight salivary cortisol 61.95 and 33.04 nmol/L (reference range 0.00–10.40 nmol/L) and loss of normal diurnal rhythm (morning plasma cortisol 924.01 nmol/L and midnight plasma cortisol 959.39 nmol/L). Further inspections were consistent with the diagnosis of adrenocorticotropic hormone (ACTH)-independent CS: 1 mg desamethasone overnight suppression test (DST) showed lack of cortisol suppression, along with repeated findings of undetectable serum ACTH concentrations (< 5 pg/ml at 8:00 AM, reference range 0–46 pg/ml). Magnetic resonance imaging (MRI) scanning without contrast revealed a left adrenal nodule measuring 2.0 cm in diameter (Fig. 1), while pituitary MRI scanning was normal. Contrast on hormonal status at each stage of the patient was shown in Table 1.

**Table 1:** Contrast on hormonal status at each stage of the patient

Laboratory assessment	Week 28 of pregnancy	Week 35 of pregnancy	3 days after parturition	8 weeks after parturition
24 h UFC/(nmol/24 h)				
Reference range	153.2–789.4	153.2–789.4	153.2–789.4	153.2–789.4
Day 1	2611.0	4808.0	1650.0	723.5
Day 2	2590.0	4598.0	–	547.0
Midnight Salivary Cortisol/(nmol/L)				
Reference range	0.00–10.40	0.00–10.40	0.00–10.40	0.00–10.40
Day 1	61.95	51.00	–	9.75
Day 2	33.04	36.86	–	–
Diurnal rhythm				
Reference range (Plasma Cortisol at 8:00 AM)	118.60–618.00	118.60–618.00	118.60–618.00	118.60–618.00
Day 1: 8:00 AM- 4:00 PM- 12:00 PM	924.01–733.26-959.39	779.05–840.11-1010.46	538.24–479.18-537.80	314.46–236.28-224.07
DEXA, 1 mg, 12:00 PM				
Day 2: 8:00 AM	872.34	–	–	286.64
Serum ACTH/(pg/ml)				
Reference range	0–46	0–46	0–46	0–46
8:00 AM	< 5	< 5	9	< 5

*24 h UFC* 24 h free urinary cortisol, *DEXA* desamethasone, *ACTH* adrenocorticotropin

Our patient did not receive specific treatment of hypercortisolism and a conservative treatment strategy was conducted. The maintenance of pregnancy was under close monitoring of blood pressure and satisfactory management of blood glucose by insulin. Sylvite supplementary treatment was adopted to remedy hypokalemia. At 35 weeks GA, her cortisol level displayed a tendency to rise up with elevated 24 h UFC reaching to 4808.0 nmol/24 h. At the request of the patient and her family, the patient underwent vaginal trial production at 36 weeks GA. Given that she developed worsening hypertension (blood pressure 154/103 mmHg) under the application of oxytocin for hastening parturition, a caesarean operation was performed and a live female infant was delivered (weighing 2820 g, 48 cm in length, Apgar 10 at 1 min, 10 at 5 min). The infant suffered from hypoglycemia and required admission to the neonatal unit. At 3 days after parturition, the plasma cortisol level plummeted to normal, but elevated 24 h UFC and the absence of normal diurnal rhythm still existed. Of note, the serum ACTH rose up slightly (9 pg/ml at 8 am). At 5 days after parturition, the woman and the infant discharged from hospital in good condition with no clinical evidence of adrenal insufficiency.

**Fig. 2:**
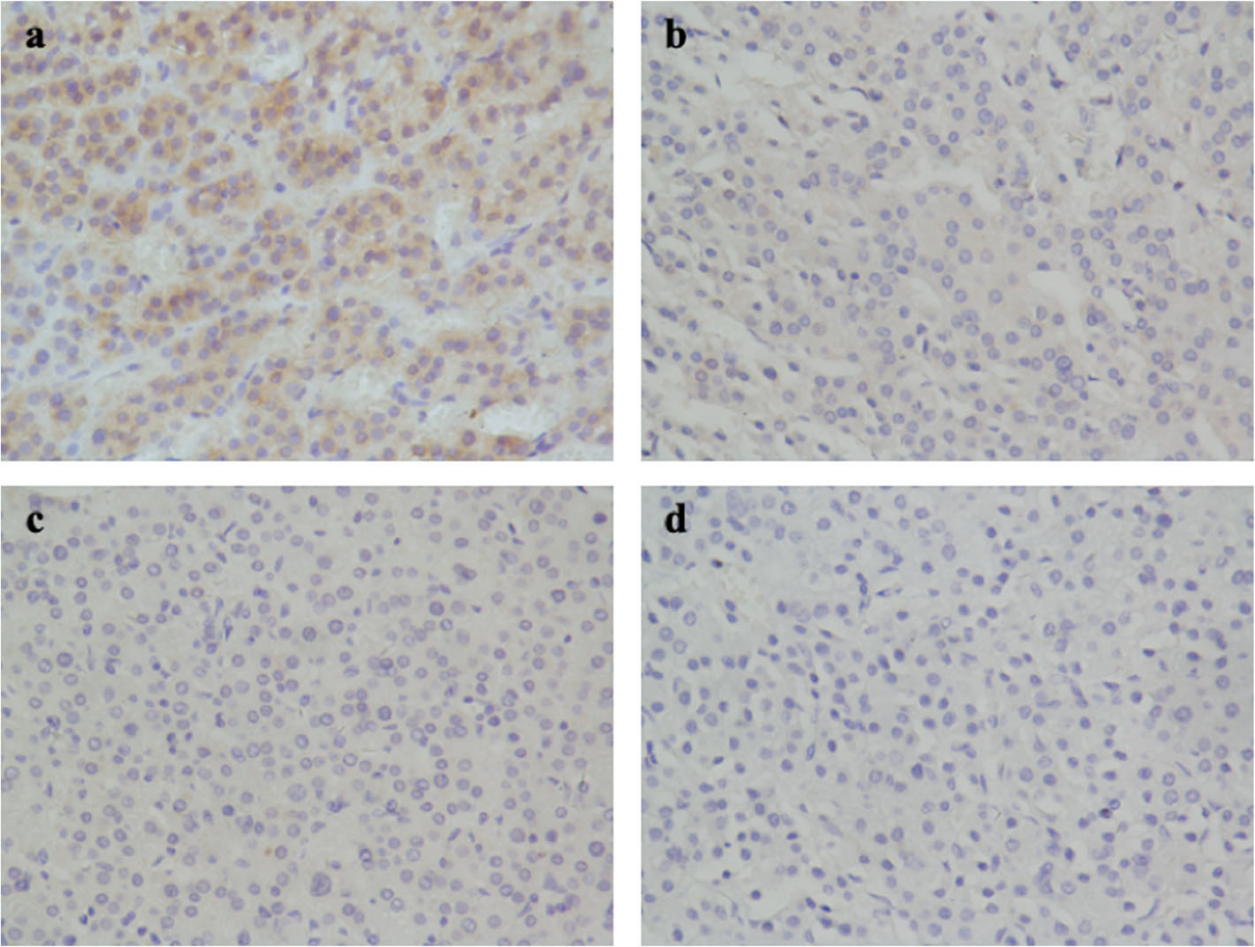
Immunohistochemical findings, magnification × 400. Immunohistochemistry showed the adrenal adenoma tissue that stained positive for LHCGR (**a**), and negative for MC2R (**b**), GPER-1 (**c**). Negative controls omitted primary antibody (**d**)

At 8 weeks after parturition, our patient achieved normalization of blood pressure, blood glucose, serum potassium, and cortisol level spontaneously. However, loss of normal diurnal rhythm, lack of cortisol suppression by DST and undetectable serum ACTH remained. At 6 months post-partum, stimulation testing with exogenous hCG (10,000 IU) elicited increased cortisol level (basal plasma cortisol 287.69 nmol/L increased to 532.99 nmol/L during the test, as shown in Table 2). As scheduled, the woman underwent resection of the adrenal tumor and routine glucocorticoid supplementation was conducted in post operation period. IHC was performed on the tumor tissue to detect the expression of LHCGR, MC2R and GPER-1 (Fig. 2).

**Table 2:** The result of stimulation testing with exogenous hCG

Time post hCG Administration (hours)	Serum hCG (IU/L)	Plasma Cortisol (nmol/L)	Serum ACTH (pg/ml)
0	4.82	287.69	< 5
8	85.68	448.69	< 5
24	116.36	458.90	< 5
36	79.93	532.99	< 5
48	67.60	435.82	< 5
72	37.34	350.62	6

*hCG* human choriogonadotropin

## Discussion and conclusions

Transient pregnancy-induced CS is quite rare and 15 cases were reported in the world literature to our knowledge [1–15]. Symptoms and signs of hypercortisolism only arise during pregnancy and remit spontaneously after delivery or abortion. This peculiar disorder challenges the canonical diagnosis of CS due to changes in the hypothalamic-pituitary-adrenal (HPA) axis during pregnancy [13] and provides a unique insight into the underlying molecular pathogenesis of adrenal pathological alterations subsequent to aberrant activation of specific receptors.

Despite the marked clinical symptoms and physical signs of hypercortisolism, it is hard to distinguish CS from nonpathologic hypercortisolism during normal pregnancies. The up-regulated HPA axis function in pregnancy is associated with placental ACTH and the increased hepatic synthesis of corticosteroid-binding globulin (CBG) stimulated by elevated circulating estrogens. Compared to nonpregnancy controls, the plasma cortisol in pregnancy is 2- to 3- fold increased, while mean 24 h UFC is elevated at least 180% [18]. Since UFC excretion is normal in the first trimester, only up to 2- to 3-fold the upper limit of normal values of UFC in the second or third trimester is recommended as a diagnostic indicator for CS in pregnant women [19]. Meanwhile, loss of normal diurnal rhythm remains reliable as a marker of CS during pregnancy, for the circadian rhythm of cortisol is preserved in normal pregnancy. Moreover, salivary cortisol level should be considered as a meaningful criterion to identify CS in pregnancy in the initial testings [20], because there is no significant variation in salivary cortisol during pregnancy, albeit in a small number of women evaluated. Whereas, DST has an increasing potential to be false-positive because the response of cortisol level to dexamethasone is blunted in pregnancy [18]. In our case, serum ACTH level was continuously undetectable until 3 days post-partum it bounced to 9 pg/ml, whereas it was still undetectable even 8 weeks post-partum. Typical manifestations with more than 3-fold increase of 24 h UFC, a 5-fold increase of midnight salivary cortical, no response to DST and the suppressed ACTH levels made the diagnosis of ACTH-independent CS unequivocal. Furthermore, the notable withdrawal of hypercortisolism after parturition and the positive stimulation testing with exogenous hCG injection were explicit diagnostic characteristics of hCG-mediated hypercortisolism.

Although rare, CS in pregnancy is associated with significant maternal and fetal complications [21] and the management of this disorder remains challenging. Owing to its safety and effectiveness, surgical management, ideally during the second trimester [13], has been recommended as the most favorable option for treatment of both pituitary and adrenal CS during pregnancy [22]. If surgery is contraindicated or must be delayed, treatment with steroidogenesis inhibitors, usually with metyrapone, can be an alternative [22]. Metyrapone has been shown to control hypercortisolism effectively in most instances [23], despite its adverse effects including hypertension, worsening of preeclampsia and the risk of affecting fetal adrenal steroid synthesis since it can cross the placenta [24]. In our case, the patient was managed conservatively by controlling Cushingoid comorbidities and achieved satisfactory management of blood pressure and blood glucose, given that the adrenal mass was determined by MRI late in the third trimester. After parturition, the woman underwent resection of the adrenal tumor. Therefore, the proper treatment strategy for the management of this rare condition should be individualized for each patient to improve outcome for both mother and fetus.

In the literature, we found two other cases of pregnancy-induced CS with a cause of an adrenal tumor overexpressing LHCGR [12, 14]. In our case, IHC staining for LHCGR on the adrenal adenoma tissue also revealed high intensity, which revalidates the essential role of LHCGR in this particular condition. Recently, the cAMP pathway has been recognized as the functional pathway downstream to LHCGR in pregnancy-induced CS. Anne Trinh et al. [14] described a case developing Cushing’s syndrome in pregnancy with an adrenal tumor harboring both LHCGR expression and a codon 201 mutation in the G alpha subunit in the cAMP pathway (GNAS), and characterized the underlying molecular pathogenesis as aberrant amplification of cAMP signaling pathway subsequent to LHCGR overexpression. Furthermore, in a case of pregnancy-induced CS with bilaterally enlarged adrenals reported by Plockinger U et al., hCG stimulation of cultured primary adrenal cells overexpressing LHCGR significantly elicited increased cAMP production and resulted in glucocorticoids synthesis, suggesting the possible role of LH/hCG-stimulated transformation of LHCGR-positive undifferentiated subcapsular cells (presumably adrenocortical progenitors) into LHCGR-positive hyperplastic cortical cells responding to LH/hCG stimulation with ACTH-independent hormonal production [15]. Taken together, these findings indicated the essential role of the aberrant activation of LHCGR coupled to an up-regulated cAMP pathway in the pathophysiology of pregnancy-induced CS. Unlike the constitutive activation of cAMP/PKA signaling due to a high frequency (35–65%) of somatic mutations affecting PRKACA in CPAs [16, 17], LHCGR-mediated signaling may serve as an exceptional way to activate the cAMP pathway, leading to hCG-dependent cortisol excess. A reasonable assumption is that persistently increased hCG level in early pregnancy activates aberrantly upregulated LHCGR, subsequently triggers the downstream signal transduction, and thus enhances the production of glucocorticoids. After delivery, when endogenous cortisol secretion returned to normal, the significant cortisol increase evoked by exogenous hCG during non-pregnancy state indicated the positive response of LHCGR to hCG stimulus.

ACTH is the principal regulator of adrenal cortisol production and signals through reacting on its receptor, MC2R. MC2R, a highly expressed GPCR on the surface of normal adrenocortical cells which binds one sole ligand-ACTH [17], was negative for IHC staining in our case, suggesting that MC2R may not be required for the excessive cortisol secretion in this condition. Considering that elevated estrogen during pregnancy could also potentially contribute to the activation of cAMP pathway through reacting on corresponding GPCR-GPER-1, IHC was performed on the tumor tissue to detect the expression of GPER-1 and the staining for GPER-1 was also negative. As a recently identified GPCR, GPER-1 has been reported to modulate aldosterone synthesis in aldosterone producing adenoma cells [25], but whether GPER-1 plays a role in the excessive hormone production and tumor formation of CPA remains to be elucidated.

In conclusion, we describe a case of pregnancy-induced CS with an adrenal adenoma overexpressing LHCGR and discuss its underlying pathogenetic mechanism on the basis of previous findings and our IHC results. Stimulation test with exogenous hCG after parturition is necessary to identify pregnancy-induced CS. LHCGR-mediated activation of cAMP signaling pathway may serve as an essential role in the pathogenesis of this particular condition.

## Data Availability

All data generated or analysed during this study are included in this published article [and its supplementary information files].

## Abbreviations

24 h UFC: 24 h free urinary cortisol
ACTH: Adrenocorticotropic hormone
cAMP: Cyclic adenosine monophosphate
CBG: Corticosteroid-binding globulin
CPA: Cortisol-producing adrenal adenoma
CS: Cushing’s syndrome
DEXA: Desamethasone
DST: Desamethasone overnight suppression test
GA: Gestational age
GDM: Gestational diabetes mellitus
GPCR: G-protein-coupled receptor
GPER-1: G protein-coupled receptor-1
GNAS: G alpha subunit in the cAMP pathway
hCG: Human choriogonadotropin
HPA axis: Hypothalamic-pituitary-adrenal axis
IHC: Immunohistochemistry
LH: Luteinizing hormone
LHCGR: Luteinizing hormone/human choriogonadotropin receptor
MC2R: Melanocortin 2 receptor
MRI: Magnetic resonance imaging
OGTT: Oral glucose tolerance test
PKA: Protein kinase A
PRKACA: cAMP dependent protein kinase A
